# High-Throughput 16S rRNA Gene Sequencing of Butter Microbiota Reveals a Variety of Opportunistic Pathogens

**DOI:** 10.3390/foods9050608

**Published:** 2020-05-09

**Authors:** Mikhail Y. Syromyatnikov, Anastasia V. Kokina, Sergey A. Solodskikh, Anna V. Panevina, Evgeny S. Popov, Vasily N. Popov

**Affiliations:** 1 Department of Genetics, Cytology and Bioengineering, Voronezh State University, 394018 Voronezh, Russia; nastenka.kokina@mail.ru (A.V.K.); s.solodskih@gmail.com (S.A.S.); anna-panevina@yandex.ru (A.V.P.); pvn@bio.vsu.ru (V.N.P.); 2 Laboratory of Metagenomics and Food Biotechnology, Voronezh State University of Engineering Technologies, 394036 Voronezh, Russia; 3 Department of Service and Restaurant Business, Voronezh State University of Engineering Technologies, 394036 Voronezh, Russia; e_s_popov@mail.ru

**Keywords:** butter, high-throughput sequencing, Sanger sequencing, microbiota, lactic acid bacteria, opportunistic bacteria

## Abstract

Microbial contamination of dairy products with a high fat content (e.g., butter) has been studied insufficiently. No studies using modern molecular methods to investigate microbial communities in butter have been conducted so far. In this work, we used high-throughput sequencing and Sanger sequencing of individual bacterial colonies to analyze microbial content of commercially available butter brands. A total of 21 samples of commercially available butter brands were analyzed. We identified a total of 94 amplicon sequence variants corresponding to different microbial taxa. The most abundant lactic acid bacteria in butter were *Lactobacillus kefiri*, *Lactobacillus parakefiri*, *Lactococcus taiwanensis* and *Lactococcus raffinolactis*. A large amount of *Streptococcus* spp. bacteria (87.9% of all identified bacteria) was found in one of the butter samples. Opportunistic pathogens such as *Bacillus cereus* group, *Pseudomonas aeruginosa*, *Cronobacter* spp., *Escherichia coli*, *Listeria innocua*, *Citrobacter* spp., *Enterococcus* spp., *Klebsiella pneumonia* were detected. The analyzed butter samples were most strongly contaminated with bacteria from the *Bacillus cereus* group, and to a lesser extent - with *Cronobacter* spp. and *Enterococcus* spp. The plating and Sanger sequencing of individual colonies revealed the presence of *Enterobacter cloacae* and *Staphylococcus epidermidis*. The Sanger sequencing also showed the presence of *Cronobacter sakazakii* in butter which can be dangerous for children under the age of 1 year. We demonstrated that butter is a good growth medium for opportunistic pathogenic bacteria. Our data indicate that despite the fact that butter is a dairy product with a long shelf life, it should be subjected to quality control for the presence of opportunistic bacteria.

## 1. Introduction

Contamination of dairy products with pathogenic microorganisms, such as *Listeria monocytogenes*, *Campylobacter jejuni*, *Staphylococcus aureus* and *Salmonella* spp. is often a cause of serious digestive system disorders [1]. Dairy products have a short shelf life, because they serve as an excellent growth medium for a wide range of microbes. Consumption of dairy products contaminated with *Listeria* spp., *Salmonella* spp., *Escherichia coli*, *Campylobacter* spp., *Shigella* spp., and mycotoxin-producing fungi [2] can result in fever, nausea, vomiting, diarrhea, and abdominal pain.

Dairy products are one of the main causes of food poisoning in humans. Thus, 46 cases of gastroenteritis related to the consumption of raw milk have been registered in the USA from 1973 till 1992. In most of these cases, raw milk was contaminated with *Campylobacter* spp., *Salmonella* spp., and *E. coli* 0157:H7 [3]. Other microbial species found in raw milk are *Yersinia enterocolitica* [4] and *L. monocytogenes* [5]. Dairy products are often spoiled by *Bacillus* spp., *Pseudomonas* spp., *Lactococcus* spp. [6] and *Streptococcus* spp. [7]. The gut-associated genera *Prevotella*, *Ruminococcus*, *Bacteroides*, *Rikenella* and *Alistipes* were detected in milk samples [8].

Microbial contamination of dairy products with a high fat content (e.g., butter) has been studied insufficiently. Various pathogenic and opportunistic pathogens, such as psychotrophic bacteria, molds, yeast, coliforms, fecal coliforms, *E. coli*, and *S. aureus*, have been found in cooking butter [9]. Analysis of butter made of goat milk revealed the presence of lactic acid bacteria, psychotrophic bacteria, molds, yeasts, and lipolytic bacteria (as well as coliforms in one of the samples) [10]. Studies of bacterial communities in petroleum oil in stockpiles in Japan based on 16S rRNA gene sequencing showed the presence of *Ochrobactrum anthropi*, *Burkholderia cepacia*, *Stenotrophomonas maltophilia*, *Propionibacterium acnes*, and *Brevundimonas diminuta* [11]. Analysis of microbial content of traditional butters produced in the West Azerbaijan province of Iran identified coliforms, molds, and yeast in the assayed samples. Thus, *S. aureus* and *E. coli* were found in 8.3 and 50% of samples, respectively [12]. Identification of microbial contaminants in commercially available butters in Turkey revealed the presence of *Enterococcus faecalis* followed by *Staphylococcus hominis*, *Micrococcus luteus*, *Pseudomonas putida*, *P. fluorescens*, *Brevibacillus brevis*, *Streptococcus sanguis*, *Weissella viridescence*, *Lactococcus lactis*, and *Lactobacillus kefir*, as well as molds and yeast [13]. In mayonnaise, another high-fat product, the yeast *Pichia kudriavzevii* was found [14].

It should be noted that these few cited works have used classical microbiological approaches (except the work of Yoshida et al. [11]) to survey butter or oil for microbial contamination. We believe that the use of modern methods of high-throughput sequencing might considerably expand our knowledge on microbial communities present in food with a high fat content. NGS (next generation sequencing) can be used as a new approach in food quality control for identification of microorganisms directly in food samples [15,16].

Studies on the microbial contamination of products with a high fat content are scarce, probably, due to the fact that such products have a relatively long shelf life and the high fat content is commonly misconceived as a factor preventing rapid bacterial growth. Moreover, identification of butter-contaminating microbiota requires preliminary sample processing, which complicates its analysis. No studies using modern molecular methods to investigate microbial communities in butter have been conducted so far. In this work, we used high-throughput sequencing and Sanger sequencing of individual colonies to analyze microbial content of commercially available butter brands.

## 2. Materials and Methods

### 2.1. Samples

Different brands of unsalted butter from pasteurized milk cream (both Russian and foreign) available on the Russian market were studied. For analysis, 1 g of butter was used.

### 2.2. Plating and Microbial Enrichment

To evaluate microbial contamination of the analyzed butter samples, the samples were plated on universal sterile solid media—fish peptone agar (FPA) (State Scientific Center of Applied Microbiology and Biotechnology, Obolensk, Russia) containing 12 g/L fish meal hydrolysate; 12 g/L peptone, 6 g/L NaCl, and 10 g/L agar (pH 7.1–7.5).

For high-throughput sequencing, microbial cultures were enriched by growing in broth sterile medium containing only 2% glucose (Dia-M, Moscow, Russia) and 1% peptone (State Scientific Center of Applied Microbiology and Biotechnology, Obolensk, Russia). Food substrate (1 g of butter) was cut into 5 to 7 mm pieces and added to 9 mL of the medium, and the microorganisms were grown for 24 h at 27 °C.

### 2.3. DNA Isolation

DNA from microbial colonies for Sanger sequencing or cells grown in the enrichment medium for high-throughput sequencing was isolated using ZymoBIOMICS DNA Kit (Zymo Research, Irvine, CA, USA).

### 2.4. DNA Barcoding

PCR was performed on individual bacterial colonies using a ScreenMix-HS reaction mixture (Evrogen, Moscow, Russia) in an Eppendorf MasterCycler Personal cycler. Direct and reverse primers for bacterial DNA amplification were 785F (5′-GGATTAGATACCCTGGTA) and 1492R (5′-TACGGYTACCTTGTTACGACTT) [17], respectively. PCR was performed in the following regime: Denaturation at 94 °C for 4 min, 35 cycles of 94 °C for 30 s, 52 °C for 30 s, and 72 °C for 45 s, and final elongation at 72 °C for 10 min. Amplified fragments were isolated from the gel and purified with a Cleanup Standard kit (Evrogen, Moscow, Russia). Purified PCR products were sequenced with an Applied Biosystems 3500 sequencer using a BigDye Terminator v3.1 Cycle Sequencing Kit, and obtained nucleotide sequences were analyzed using the GenBank database. Organisms were assigned to certain taxa if their DNA sequences coincided by no less than 99% with the corresponding sequences already deposited in the databases by at least five different authors.

### 2.5. High-Throughput Sequencing

The microorganisms were collected from the enrichment broth medium by centrifugation at 10,000× *g* for 5 min. Bacterial DNA isolated from collected bacteria was amplified with the universal direct 785F forward primer (5′-GGATTAGATACCCTGGTA) and reverse 1100R primer (5′-GGGTTGCGCTCGTTG) [17]. PCR was performed using a ScreenMix-HS Master Mix (Evrogen, Moscow, Russia) in the following regime: 94 °C for 4 min followed by 37 cycles of 94 °C for 30 s, 53 °C for 30 s, and 72 °C for 30 s, with the final elongation at 72 °C for 5 min. PCR products were purified with AMPure XP magnetic beads (Beckman Coulter, Miami, FL, USA) and used for the construction of sequencing libraries using Ion AmpliSeq Library Kit 2.0 (Thermo Fisher Scientific, Waltham, MA, USA) as recommended by the manufacturer. Barcoding was done using the Ion Xpress barcode adapters (Thermo Fisher Scientific, Waltham, MA, USA). Library DNA concentration was determined by qPCR using Library Quantification Kit Ion Torrent Platforms (Kapa Biosystems, Wilmington, MA, USA).

Sequencing was performed with the IonTorrent PGM platform using Ion PGM Hi-Q View Sequencing Kit, Ion OneTouch 2 System, and Ion PGM Hi-Q View OT2 Kit (Thermo Fisher Scientific, Waltham, MA, USA).

The results of sequencing were obtained as binary alignment map (BAM) files that were converted into FASTQ format using the SAMtool v.1.2 software. Demultiplexing and primer stripping was done using the fastq-multx application of the ea-utils v.1.3. program package. The reads were then filtered according to the reading quality based on the number of expected errors [18,19].

Unique sequences were identified using the DADA2 package version 1.8.0. We used the negative homopolymer gap penalty value (parameter HOMOPOLYMER_GAP_PENALTY = −1), which causes homopolymer gaps to be treated as homopolymer sequences, and increased net cumulative number of insertion of one sequence relative to the other (parameter BAND_SIZE = 32).

After that, we constructed an amplicon sequence variant (ASV) table and filtered out chimeric sequences. Taxonomy (with genus-level resolution) was assigned to the sequence variants using DADA2 implementation of the naive Bayesian classifier method [20]. Species level taxonomy was assigned using exact matching (100% identity) with amplicon sequence variants. Both genus and species identification were performed with the SILVA database (https://www.arb-silva.de) version 132 as a reference. We used R version 3.4.4 for all operations related to NGS data analysis and taxonomy assignment.

## 3. Results

We analyzed 21 samples of commercially available brands. Since isolation of microbial DNA directly from the samples was impossible because of the high fat content of butter, we enriched the samples by incubation in liquid nutritive medium. DNA was amplified by PCR and subjected to high-throughput sequencing followed by bioinformatics analysis. Raw sequencing data is available from NCBI Sequence Read Archive (SRA), unique ID PRJNA526897.

Using DADA2 algorithm—which produces amplicon sequence variants (ASVs) or zero-radius OTUs (operational taxonomic units), instead of OTUs produced by traditional methods—we identified a total of 108 ASVs. After taxonomy assignment, we filtered out 14 ASVs that did not match any records in the reference database. The remaining 94 ASVs were identified up to genus level, and 85 of them were identified up to species level. In this paper, we report species of the microorganism only if there were no ambiguity during taxonomy identification (i.e., all exact matches of the ASV sequence were to the reference sequences of the same species). In all other cases, genus of the microorganism is reported.

The prevalence of particular bacterial species varied in the samples. The reads were unevenly distributed among different bacterial taxa. We estimated the total abundance of all identified taxa based on the number of reads. The most abundant bacteria for all samples are shown in Table 1. See the supplemental materials for the ASVs of “Minor species” for all butter samples.

**Figure 1:**
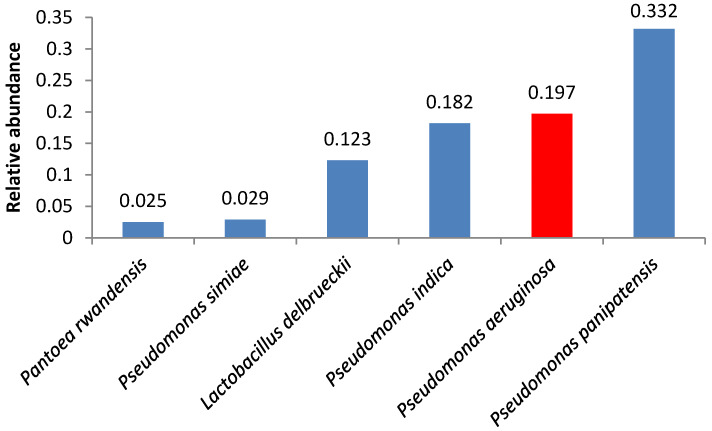
Relative abundance of amplicon sequence variants in butter sample no. 4.

**Figure 2:**
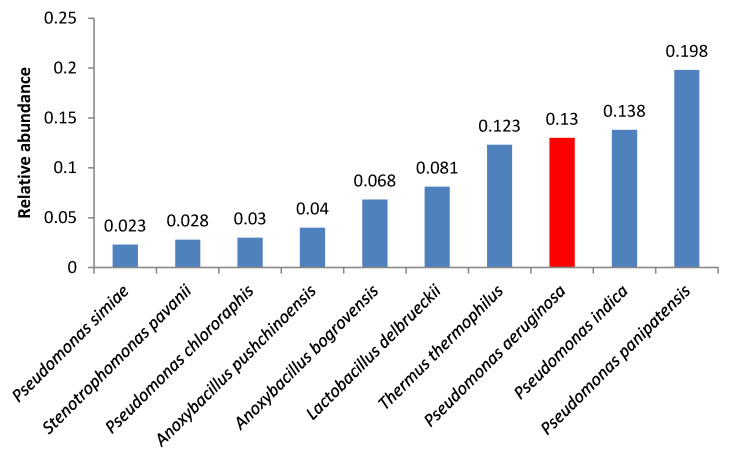
Relative abundance of amplicon sequence variants in butter sample no. 2.

A number of samples contained opportunistic bacteria; in some of these samples, the amount of these bacteria was relatively high. *Pseudomonas aeruginosa* was the second and the third most abundant microorganism in samples 4 and 2, respectively (Figure 1 and Figure 2).

**Figure 3:**
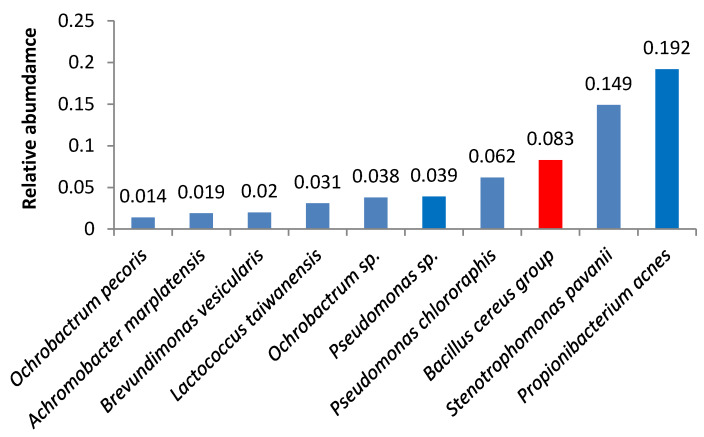
Relative abundance of amplicon sequence variants in butter sample no. 3.

**Figure 4:**
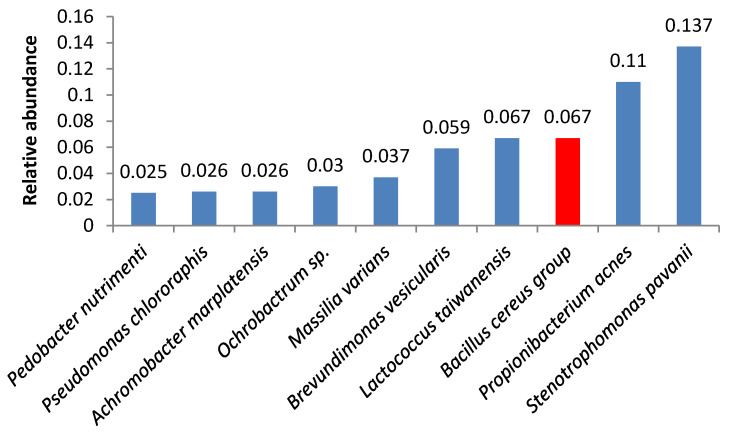
Relative abundance of amplicon sequence variants in butter sample no. 18.

Bacteria of the *Bacillus cereus* group were the third most abundant microbes in samples 3 and 18 (Figure 3 and Figure 4). Samples 1, 6, 16, 5, 19, and 21 also contained high amounts of *Bacillus cereus* group bacteria.

**Figure 5:**
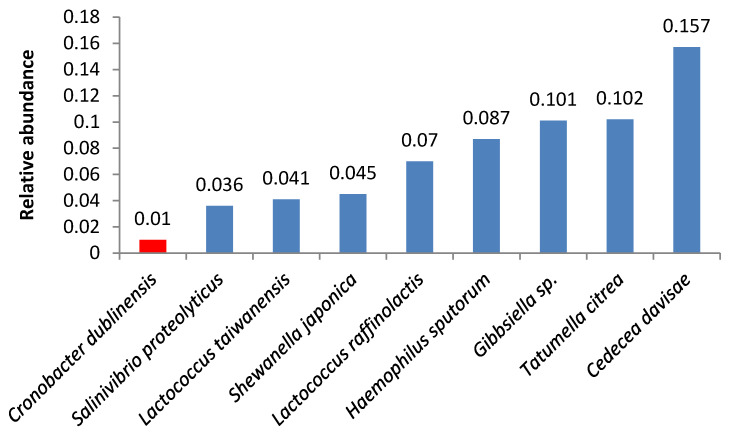
Relative abundance of amplicon sequence variants in butter sample no. 8.

**Figure 6:**
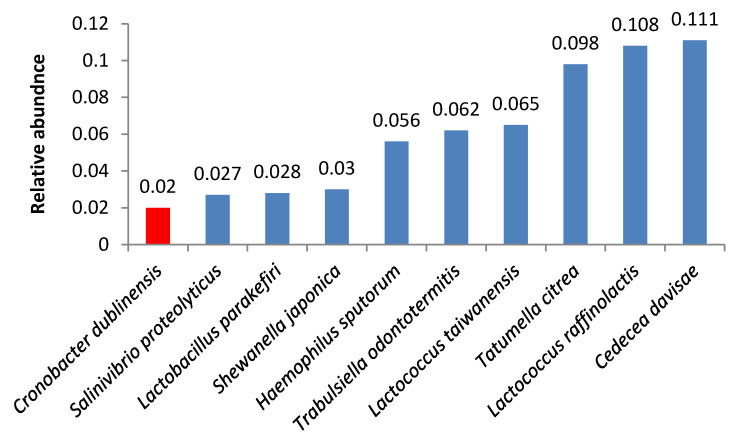
Relative abundance of amplicon sequence variants in butter sample no. 12.

We also found other opportunistic pathogens. Samples 8 and 12 contained high quantities of bacteria from the *Cronobacter* genus (Figure 5, Figure 6); the same species were identified in smaller amounts in samples 7 and 9.

*Enterococcus* sp. bacteria were found in samples 10, 13, and 19. *E. coli* and *Klebsiella pneumoniae* were found in three samples each. Six samples contained bacteria of the *Enterobacter* genus; one sample (no. 6) was contaminated with *Citrobacter* sp. bacteria. Sample 12 contained *Listeria innocua*. Bacteria of the *Streptococcus* and *Staphylococcus* genera were identified in almost all analyzed butter samples. All the aforementioned bacteria were considered to be present in the samples when the number of reads per ASV exceeded 500. However, the probability of ASV identification for a particular microorganism varied depending on the threshold value for the number of reads per ASV (Table 2).

The analyzed butter samples were most strongly contaminated with bacteria from the *Bacillus cereus* group, and to a lesser extent, with *Cronobacter* spp. and *Enterococcus* spp. No dangerous foodborne pathogens, such as *Salmonella* spp. and *L. monocytogenes*, were found.

Samples containing dangerous pathogenic bacteria were plated onto universal nutritive medium (see Section 2 Materials and Methods), and the grown colonies were used for Sanger sequencing of the 16S rRNA gene fragment. Results are shown in Table 3.

Using the method of sequencing of individual bacterial colonies, opportunistic bacteria were identified in butter samples, such as *Cronobacter sakazakii*, *Bacillus thuringiensis* (*Bacillus cereus* group) and *Enterobacter cloacae*.

Butter samples also contained a high amount of lactic acid bacteria. Relative abundance of lactic acid bacteria (order Lactobacillales) in all butter samples is presented in Table 4.

Samples 11 and 14 contained a large number of bacteria of *Lactobacillus* genus (*Lactobacillus kefiri* and *Lactobacillus parakefiri*)—overall relative abundance, 66.8% and 68.7%, respectively. Samples 16, 17 and 20 contained a large number of bacteria *Lactococcus taiwanensis*—relative abundance, 14.2%, 71.3% and 34.2%, respectively. In addition, sample 20 contained *Lactococcus raffinolactis* in high abundance (46.6%). Sample 13 contained a large number of bacteria of *Streptococcus* genus (overall genus relative abundance, 87.9%). For the relative abundance of lactic acid bacteria ASV (expressed in percentage) in butter samples, see the supplemental materials.

## 4. Discussion

In this work, we studied bacteria contaminating Russian and foreign brands of butter available on the Russian market. The microbial communities in butter samples were very diverse and included many opportunistic pathogens, as demonstrated by high-throughput sequencing. The advantage of this method, in comparison to classical microbiological and immunological approaches, as well as PCR, is the possibility of simultaneous sequencing of DNA fragments from all the bacteria present in the sample. We used the universal primer pair 785F/1100R (see Section 2 Materials and Methods) to amplify a fragment of the 16S rRNA gene including the V4, V5, and V6 regions that have been demonstrated to be the most efficient hypervariable regions for phylogenetic analysis and taxonomic classification of bacterial species [21].

For over a decade, OTUs with 97% identity threshold were used for the identification of microorganisms using 16S rRNA gene sequencing. Recently, it was proposed that exact sequence variants (also referred to as zero-radius OTUs or OTUs with 100% identity) can be used instead of 97% threshold operational taxonomic units in metagenomic studies [22]. Later, it was shown that ASVs demonstrate higher accuracy and lower error rates in species-level classification compared to OTUs both in mock communities [23] and in research applications [24,25] while decreasing the number of identified taxa. In this study, we used the DADA2 algorithm for the identification of ASVs.

We used naïve Bayesian classifier for the identification of microorganisms up to the genus level. Recently, it was shown that species-level identification is possible only if 100% identity threshold is used for V4 hypervariable region of 16s rRNA gene [26]. As we used the V4–V6 hypervariable region, we decided to use 100% identity for species-level classification. Species-level classification was performed using exact string matching against a reference database (SILVA v. 132).

Since it is almost impossible to isolate DNA directly from bacteria in butter samples because of the high fat content in butter, we first enriched the bacteria in liquid nutritive medium. It is important to remember that enrichment can change the relative content of individual bacterial species in the sample. At the same time, enrichment allows the contamination of the sequencing results with DNA from dead bacteria to be minimized, so long as the number of live bacteria in the sample after enrichment is significantly higher than the number of dead bacteria. Therefore, ASVs corresponding to dead bacteria can be filtered out based on the low relative abundance. This is important, since detection of DNA from dead microbes is a drawback of the use of molecular-genetic methods for bacterial identification. We have decreased this possibility, because cultivation in the nutritive medium resulted in the propagation of only live bacteria that were then collected by centrifugation and used for DNA isolation.

Butter contained a large number of lactic acid bacteria. The most abundant bacteria were *Lactobacillus kefiri*, *Lactobacillus parakefiri*, *Lactococcus taiwanensis* and *Lactococcus raffinolactis*. A large amount of *Streptococcus* spp. bacteria (87.9% of all identified bacteria) was found in one of the butter samples (no. 13). However, the number of lactic acid bacteria dominated other bacteria in only 5 of the 21 samples.

We have identified different species of bacteria from the genus *Bacillus*, *Pseudomonas*, *Lactococcus* and *Streptococcus*. This is consistent with previous data for dairy products [6,7]. However, we did not find the most dangerous representatives of foodborne bacteria, such as *Salmonella* spp. and *L. monocytogenes*. Interestingly, some of the bacteria, such as *Ochrobactrum* sp., *Stenotrophomonas* sp., *Propionibacterium acnes*, and *Brevundimonas* sp., that was found in the commercial butter matched those found in petroleum oil earlier [11].

The analyzed butter samples were contaminated with bacteria of the *Bacillus cereus* group that includes at least 8 closely related species: *Bacillus cereus*, *B. anthracis*, *B. pseudomycoides*, *B. mycoides*, *B. weihenstephanensis*, *B. toyonensis*, *B. cytotoxicus* and *B. thuringiensis* [27,28]. We were not able to distinguish between these three species based on the sequence of the analyzed DNA fragment (unpublished data). Bacteria of the *Bacillus cereus* group can cause severe food poisoning [29], including diarrhea (due to production of thermolabile enterotoxin during propagation in small intestine) [30] or vomiting (due to secretion of the thermostable peptide toxin cereulide) [31]. The prevalence of these microorganisms in the samples was very high. Thus, the *Bacillus cereus* group was the third most abundant group of microorganisms in two butter samples. Note that the presence of these bacteria was confirmed by plating the samples onto solid medium, followed by the isolation of the grown colonies and the sequencing of the 16S rRNA gene fragment.

Two samples contained *Pseudomonas aeruginosa*; in one of them, this bacterium was the second most abundant microorganism. *P. aeruginosa* possesses an array of virulence factors counteracting host defenses and is one of the most common microbes causing respiratory infections in patients in hospitals [32]. Infections caused by *P. aeruginosa* are difficult to cure because of this microorganism’s ability to acquire resistance to antibiotics [33].

To our surprise, we identified bacteria of the *Cronobacter* genus in several butter samples by high-throughput sequencing. Sanger sequencing of bacterial colonies grown after plating of these samples on the universal nutritive medium identified them as *Cronobacter sakazakii*. *C. sakazakii* causes serious infections, such as necrotic enterocolitis, meningitis, and sepsis in formula-fed infants under 1 year of age [34]. Neonatal infections caused by *C. sakazakii* are often epidemiologically linked to the consumption of contaminated powdered infant formula [35]. It is known that *C. sakazakii* can live in low-moisture foods [36] and has high tolerance to elevated temperatures [37], which explains the presence of *Cronobacter* bacteria in dried food products. *Cronobacter* spp. have been found in powdered milk, spices, fresh vegetables, and pasta [38,39]. Here, we demonstrated for the first time, the presence of *Cronobacter* spp. in butter, which might be explained by the high tolerance of these microorganisms to media with low water content. Also, it may indicate that powdered milk could be used for the production of butter.

Identification of opportunistic microorganisms using high-throughput sequencing requires further steps to improve the methodology. We have shown that the detection of certain species of bacteria depends on the amount of reads on ASV (Table 2). It is necessary to optimize the method for detecting dangerous microorganisms in food to avoid false identification of bacteria that is dangerous to humans.

In conclusion, we demonstrated for the first time, that butter is a good growth medium for opportunistic pathogens. Bacteria from a broad range of taxa were present in butter samples in very high amounts. We were able to identify the contaminating species by both high-throughput sequencing and traditional plating on nutritive medium. Our data indicate that despite the fact that butter is a dairy product with a long shelf life, it should be subjected to quality control for the presence of pathogenic microorganisms. Based on the fact that the bacteria identified by us in the butter partially coincided with the data on the bacterial contaminant of dairy products [6,7], milk powder [35] and oil [11], we suggest that opportunistic bacteria was able to get into the butter, along with the ingredients necessary for its making. The possibility of contamination with *C. sakazakii* should be taken into account when feeding babies less than 1 year of age with butter-containing food products.

## Figures and Tables

**Table 1:** The most abundant bacteria in butter samples.

Bacteria	Butter Sample																				
	1	2	3	4	5	6	7	8	9	10	11	12	13	14	15	16	17	18	19	20	21
*Geobacillus* spp.																					
*Stenotrophomonas* spp.																					
*Thermus thermophilus*																					
*Cedecea davisae*																					
*Tatumella citrea*																					
*Lactobacillus* spp.																					
*Lactococcus* spp.																					
*Streptococcus* spp.																					
*Acinetobacter* spp.																					
*Anoxybacillus* spp.																					
*Xanthomonas bromi*																					
*Pseudoxanthomonas* spp.																					
*Propionibacterium acnes*																					
*Leuconostoc* spp.																					
*Pseudomonas* spp.																					
*Massilia varians*																					
*Ochrobactrum* spp.																					
*Bacillus cereus group*																					
*Chryseobacterium* spp.																					
*Trabulsiella* spp.																					
*Shewanella japonica*																					
*Haemophilus sputorum*																					
*Pantoea* spp.																					
*Gibbsiella* spp.																					
*Brevundimonas* spp.																					
<1% -no color	1–10%			21–30%			21–30%			31–40%			41–50%			51–60%			>61%		

**Table 2:** The prevalence of opportunistic bacteria at different threshold values for the number of reads per ASV.

Opportunistic Pathogen	Found in Samples, % (10–100 Reads Per ASV)	Found in Samples, % (100–500 Reads Per ASV)	Found in Samples, % (>500 Reads Per ASV)
*Bacillus cereus* group	100	66.7	52.4
*Pseudomonas aeruginosa*	38.1	23.8	9.6
*Cronobacter* spp.	57.2	42.9	14.3
*Escherichia coli*	61.9	28.6	14.3
*Listeria innocua*	38.1	14.3	4.8
*Citrobacter* spp.	14.3	9.5	4.8
*Enterococcus* spp.	90.5	66.7	14.3
*Klebsiella pneumoniae*	28.6	28.6	14.3

**Table 3:** Bacteria identified by Sanger sequencing of individual colonies.

Butter Sample	Amount of Bacteria, CFU/g	Bacteria Identified in the Sample
2	3.0 × 10^5^	*Bacillus pumilus, Acinetobacter guillouiae, Rothia* sp., *Rahnella aquatilis, Pseudomonas* sp.
3	3.0 × 10^4^	*Bacillus thuringiensis, Neisseria* sp., *Moraxella osloensis*
4	2.0 × 10^4^	*Enterococcus* sp., *Zimmermannella faecalis*, *Pseudomonas* sp.
6	5.0 × 10^4^	*Lysinibacillus* sp., *Carnobacterium maltaromaticum*, *Pseudomonas* sp.
12	7.0 × 10^5^	*Enterococcus italicus, Pseudomonas* sp., *Kocuria salsicia, Cronobacter sakazakii,*
18	1.0 × 10^6^	*Enterobacter cloacae, Staphylococcus epidermidis, Lysinibacillus* sp., *Micrococcus* sp.

**Table 4:** Relative abundance of lactic acid bacteria (order Lactobacillales) amplicon sequence variants in all butter samples.

Bacteria	Butter Sample																				
	1	2	3	4	5	6	7	8	9	10	11	12	13	14	15	16	17	18	19	20	21
*Lactobacillus kisonensis*																					
*Lactobacillus senioris*																					
*Lactobacillus diolivorans*																					
*Lactobacillus kefiri*																					
*Lactobacillus parakefiri*																					
*Lactobacillus delbrueckii*																					
*Lactococcus taiwanensis*																					
*Lactococcus chungangensis*																					
*Lactococcus plantarum*																					
*Lactococcus raffinolactis*																					
*Streptococcus infantarius*																					
*Streptococcus vestibularis*																					
*Streptococcus porcorum*																					
*Streptococcus hongkongensis*																					
*Leuconostoc pseudomesenteroides*																					
0% -no color	<0.1%			0.1–1%			2–15%			16–30%			31–45%			46–60%			61–75%		

## Supplementary Materials

The following are available online at https://www.mdpi.com/2304-8158/9/5/608/s1, File “S1_Minor bacteria species in butter”. File “S2_Relative abundance of lactic acid bacteria (order Lactobacillales) ASV in all butter samples”.
