# Supplementary material for: High-Throughput 16S rRNA Gene Sequencing of Butter Microbiota Reveals a Variety of Opportunistic Pathogens
Source: Foods. 2020 May 9;9(5):608. doi: 10.3390/foods9050608 (PMC7278763; doi:10.3390/foods9050608)
Supplement: Supplementary file 1 [file foods-09-00608-s001.pdf]

S1_Minor bacteria ASV detected in butter samples.

1. *Ochrobactrum* sp.;
2. *Dickeya dianthicola*;
3. *Shewanella japonica*;
4. *Chryseobacterium anthropi*;
5. *Pseudomonas simiae*;
6. *Trabulsiella odontotermitis*;
7. *Kangiella aquimarina*;
8. *Salinivibrio proteolyticus*;
9. *Achromobacter marplatensis;*
10. *Streptophyta* sp.;
11. *Lactococcus plantarum*;
12. *Lactobacillus kisonensis*;
13. *Massilia varians;*
14. *Enterococcus* sp.
15. *Geobacillus caldoxylosilyticus;*
16. *Stenotrophomonas rhizophila;*
17. *Chryseobacterium haifense;*
18. *Serratia* sp.;
19. *Pedobacter nutrimenti;*
20. *Tatumella terrea;*
21. *Pseudoxanthomonas wuyuanensis;*
22. *Pseudocitrobacter* sp.;
23. *Leuconostoc pseudomesenteroides;*
24. *Anoxybacillus salavatliensis;*
25. *Staphylococcus petrasii*;
26. *Cronobacter dublinensis*;
27. *Pantoea rwandensis*;
28. *Achromobacter pulmonis;*
29. *Pseudomonas* sp.;
30. *Bacillus smithii;*
31. *Pseudomonas granadensis;*
32. *Klebsiella singaporensis;*
33. *Klebsiella pneumoniae*
34. *Brevundimonas aurantiaca;*
35. *Ochrobactrum pectoris;*
36. *Macrococcus caseolyticus;*

37. Aeromonas australiensis;
38. *Brevundimonas mediterranea*;
39. Pseudomonas prosekii;
40. Lactobacillus senioris;
41. Geobacillus icigianus;
42. *Chryseobacterium* bovis;
43. Streptococcus hongkongensis;
44. *Anoxybacillus thermarum*;
45. Aeromonas bestiarum;
46. Listeria innocua
47. *Ochrobactrum* sp.;
48. Anoxybacillus voinovskiensis;
49. *Lonsdalea quercina;*
50. Pseudomonas palleroniana;
51. Staphylococcus agnetis;
52. Micrococcus flavus;
53. Escherichia coli
54. Pedobacter panaciterrae;
55. *Citrobacter* sp.;
56. *Phyllobacterium myrsinacearum*;
57. Pseudomonas amygdale;
58. *Morganella* sp.;
59. *Geobacillus thermantarcticus*.

S2_Relative abundance (%) of lactic acid bacteria (order Lactobacillales) ASV in all butter samples.

**Bacteria**	**Butter Sample**																				
	**1**	**2**	**3**	**4**	**5**	**6**	**7**	**8**	**9**	**10**	**11**	**12**	**13**	**14**	**15**	**16**	**17**	**18**	**19**	**20**	**21**
Lactobacillus kisonensis	0	0	0	0	0	0	0	0.1	0.1	0	3.8	0.2	<0.1	4.6	0	0	0	0	0	0.1	0
Lactobacillus senioris	<0.1	<0.1	0	0	0	0	0	0.1	0.1	0	1.3	0.2	<0.1	1.7	<0.1	0	<0.1	<0.1	<0.1	0.1	<0.1
Lactobacillus diolivorans	<0.1	<0.1	<0.1	0	<0.1	<0.1	0.4	0.2	0.3	<0.1	6.7	0.4	0,2	6.5	<0.1	<0.1	<0.1	<0.1	<0.1	0.3	<0.1
Lactobacillus kefiri	<0.1	<0.1	<0.1	0	0	<0.1	0.4	0.6	0.6	<0.1	20.0	1.0	0,5	19.0	<0.1	<0.1	<0.1	<0.1	<0.1	0.7	<0.1
Lactobacillus parakefiri	<0.1	<0.1	0	0	0	0	2.2	1.7	1.8	0	46.8	2.8	1.4	49.7	<0.1	2.8	<0.1	<0.1	<0.1	1.9	<0.1
Lactobacillus delbrueckii	<0.1	8.1	<0.1	12.3	<0.1	<0.1	0	0	<0.1	0.5	0	0	<0.1	0	<0.9	0	<0.1	0.1	0.1	0	1.0
Lactococcus taiwanensis	2.0	2.6	3.1	<0.1	0.7	1.5	7.3	4.1	2.8	10.5	3.3	6.5	1.9	2.5	2.6	14.2	71.3	6.7	8.4	34.2	4.8
Lactococcus chungangensis	0	0	0	0	0	0	1.5	0.8	0.6	0	0.7	1.4	0.4	0.5	0	0	0	0	0	7.2	0
Lactococcus plantarum	0.3	0.4	0.3	<0.1	0.4	0.2	0	<0.1	<0.1	1.2	0	0	0	<0.1	0.4	1.2	2.2	0.3	2.2	0	0.4
Lactococcus raffinolactis	0.1	0.2	0.2	<0.1	0.3	0.1	9.9	7.0	4.3	0.5	5.6	10.8	2.0	3.7	0.2	0.8	1.7	0.3	1.1	46.6	0.1
Streptococcus infantarius	<0.1	0.2	<0.1	0	<0.1	<0.1	0.4	2.1	1.8	<0.1	4,0	3.1	72.3	2.1	0.2	0.4	<0.1	0.1	0.2	2.3	0.1
Streptococcus vestibularis	0.3	0.6	0.1	<0.1	0.1	<0.1	3.6	0.3	0.5	3,0	0.2	0.5	0.3	0.3	0.6	0.7	0.2	0.1	0.3	0.4	0.2
Streptococcus porcorum	<0.1	<0.1	<0.1	<0.1	<0.1	<0.1	0.4	0.5	0.4	<0.1	0.7	0.7	15.3	0.5	<0.1	0.1	<0.1	<0.1	0.1	0.4	<0.1
Streptococcus hongkongensis	0.2	0.5	0.1	<0.1	<0.1	<0.1	0	<0.1	<0.1	1.0	0	<0.1	<0.1	<0.1	0.5	0.6	0.2	0.1	0.4	<0.1	0.2

Leuconostoc pseudomesenteroides	0.1	0.1	0.3	0	0.3	0.1	0	0	0	0.3	0	0	0	0	3.1	0.2	3.2	0.6	0.4	0	0.2
